# Antibiotic consumption by Access, Watch and Reserve index in public sector of Limpopo province, South Africa: 2014–2018

**DOI:** 10.4102/sajid.v37i1.463

**Published:** 2022-10-27

**Authors:** Tiyani C. Mthombeni, Johanita R. Burger, Martha S. Lubbe, Marlene Julyan

**Affiliations:** 1 Medicine Usage in South Africa (MUSA), Faculty of Health Sciences, North-West University, Potchefstroom, South Africa

**Keywords:** antibiotic sales data, antibiotic consumption, antibiotic resistance, antibiotic stewardship, antibiotic consumption measures, ATC/DDD index, Access-to-Watch Index, AWaRe classification, Limpopo, South Africa

## Abstract

**Background:**

The World Health Organization (WHO) classified antibiotics into three categories in 2017 – Access, Watch and Reserve (AWaRe) – intending to reduce the consumption of Watch and Reserve antibiotics while increasing the use of Access antibiotics. Antibiotic consumption by AWaRe in South Africa is undetermined because of data and research scarcity.

**Objectives:**

The aim of this study was to quantify, describe and track antibiotic consumption between 2014 and 2018 in the public sector of the Limpopo province, South Africa, using the WHO’s AWaRe classification for 2021.

**Method:**

Antibiotic consumption was quantified from pharmaceutical sales data for 2014–2018 by defined daily dose (DDD) per 1000 inhabitants per day (DID) and described according to the AWaRe classification. The change in antibiotic consumption was measured by compound annual growth rate (CAGR), Access-to-Watch index (AW-I), 75% drug utilisation index (DU75%) and amoxicillin index (AI).

**Results:**

The absolute consumption of Access antibiotics decreased by a 4.0% CAGR from 3.7 DID in 2014 to 3.0 DID in 2018, with relative consumption remaining above 80.0%. Relative consumption of Watch antibiotics increased by 15.8% CAGR from 7.8% in 2014 to 19.7% by 2018. The AW-I decreased from 10.4 in 2015 to 4.1 in 2018. The AI increased from 17.8% in 2015 to 42.0% in 2018. Parenteral formulations’ DU75% comprised one Watch (ceftriaxone) and two Access (metronidazole and benzylpenicillin) antibiotics.

**Conclusion:**

In Limpopo province’s public sector, the consumption of Watch antibiotics increased, while Access antibiotics consumption decreased, as reflected by both relative consumption and the decrease in the AW-I. The determinants of the Watch antibiotics increase require research attention.

**Contribution:**

Our study addressed the paucity of surveillance and research data on antibiotic consumption in the Limpopo province, South Africa, according to the WHO AWaRe classification.

## Introduction

The World Health Organization (WHO) introduced the Access, Watch and Reserve (AWaRe) antibiotic classification system in 2017 upon the recommendation of its Expert Committee on the Selection and Use of Essential Medicines.^1^ The AWaRe classification is intended to promote the importance of optimal Watch and Reserve antibiotics use in consideration of the potential for antibiotic resistance (ABR) development and also for advocating the availability and use of Access antibiotics for universal health coverage.^1^ Globally, the consumption of Watch antibiotics increased more than antibiotics in the Access group, owing to increasessd consumption in low- and middle-income countries.^2^ The increasing antibiotic consumption (ABC) is a significant driver of ABR,^2,3,4^ thereby necessitating a global coordinated effort to combat it.^5^

The AWaRe classification is a valuable tool for tracking ABC, setting performance targets and evaluating the effects of the antimicrobial stewardship programme (ASP), aiming to optimise antibiotic use and limit ABR development.^1^ There are 87 Access antibiotics, 19 of which are considered first- or second-line empirical therapy for 26 common infectious syndromes and have a good safety profile with a low risk of causing ABR.^1^ Watch antibiotics have a higher ABR potential because of their broader spectrum of action. This group of antibiotics include most of the effective alternatives for a small number of well-defined clinical syndromes and/or agents that are at relatively high risk of selection of bacterial resistance. Therefore, their usage should be closely monitored and limited.^1^ The Reserve group consists of ‘last resort’ antibiotics used to treat patients with life-threatening infections caused by difficult-to-treat bacteria; hence, they must be constantly monitored and prioritised as stewardship programme targets to preserve their efficacy.^1^ Not-recommended antibiotics – a fourth category – was incorporated in the 2019 Essential Medicines List (EML) update.^6^ The Not-recommended antibiotics are specific fixed-dose combinations that have no valid indications for treating infectious illnesses in humans, posing a risk to ABR and patient safety.^6^ In 2021, the AWaRe classification was revised to include an additional 78 antibiotics in the Not-recommended group, raising their total to 103 and bringing the grand total to 360 – the other groups contributing 87 Access, 141 Watch and 29 Reserved.^7^

The AWaRe classification is new and evolving. Studies exclusively analysing ABC using it at a global,^2,8^ national^9,10^ and provincial^11^ level are scarce. There has been a poor representation of data from Africa on AWaRe analysis conducted at a global level.^2^ There are few studies on ABC in Africa that detail their reporting on AWaRe classification – limiting themselves to measuring ABC in each AWaRe category (relative or absolute) – without mentioning other AWaRe ABC indicators such as the amoxicillin index (AI).^12,13,14,15^ Therefore, studies describing research undertaken in Africa, including South Africa, that used the AWaRe classification exclusively to analyse ABC data are scarce.

South Africa has recently begun reporting ABC according to AWaRe classification; however, the report is limited to the national level; consequently, there is a gap in ABC reporting by AWaRe at the subnational level.^16^ As such, the objective of this study was to quantify, track and describe ABC data for the public sector in Limpopo province, South Africa, 2014–2018 and to report on it using the 2021 WHO AWaRe classification.

## Methods

### Study design

A descriptive repeated cross-sectional study was implemented by using aggregated data from a central, sub-national public health pharmaceutical warehouse in Limpopo province, South Africa, to investigate ABC using the WHO’s AWaRe classification.

### Study settings

The study setting was in the northern region of South Africa known as the Limpopo province, which has international borders with Mozambique, Botswana and Zimbabwe.^17^ The province has a land area of approximately 123.910 km^2^ and a population of approximately 6 million habitants.^17^ The ABC sales data were obtained from the LPPD, which is managed by the Limpopo Department of Health. The Provincial Department of Health (PDoH) serves the Limpopo population with health care services through its 523 (481 fixed and 42 mobile) primary health care clinics, 30 primary hospitals, five secondary hospitals, four specialised hospitals and two tertiary hospitals in the public sector.^18^

### Inclusion and exclusion criteria

The study population comprised the J01 antibiotics group according to the Anatomical Therapeutic Chemical (ATC) classification, which consists of antibacterials for systemic use, excluding antimycobacterials classified in J04.

### Data sources, variables and procedure

Antibiotic consumption sales data were retrieved from the pharmaceutical distribution database at the central pharmaceutical depot for the public sector to describe ABC patterns between 2014 and 2018 retrospectively. The authors adopted the Global Antimicrobial Resistance and Use Surveillance System (GLASS) methodology.^19^ The WHO GLASS methodology comprises two components: GLASS-antimicrobial resistance (AMR) and GLASS-AMC antimicrobial consumption.^19^ The GLASS-AMR is a surveillance platform for ABR, while GLASS-AMC aims to present a standardised and consistent approach for monitoring ABC. The standardisation facilitates tracking data trends over time, permits comparisons across different locations and may influence policy and practice on optimal antibiotic utilisation.^19^ The methodology incorporates a set of data gathering and reporting principles, including data sources (such as imports, procurement, sales, reimbursement and dispensing records), the ATC/DDD index, variables (such as population, grams and the number of packages) and reporting metrics (such as the defined daily dose [DDD]).^19^ Data were manually entered into the software (the WHO Antimicrobial Consumption tool) to calculate the ABC rate. Antibiotic consumption data were provided as the total number of packages distributed per calendar year for each antibiotic at ATC level 5 (i.e. the chemical substance of the medicine, e.g., J01XA01 for vancomycin). Moreover, the ABC tool has a provision for the population size adjustment; therefore, the ABC output was provided in the DDD per 1000 inhabitants per day (DID). In this analysis, the authors used Statistics South Africa’s total annual population^17^ of the Limpopo province, as it was not possible to determine the public sector population alone.

### Reported metrics and data analysis

The number of DDDs for each antibiotic agent was aggregated to determine the total number of DDDs at the required ATC level and adjusted for population size, based on Statistics South Africa,^17^ to determine the absolute consumption rate in DID according to equation 1.^20^

**Antibiotic consumption rate in DID**


$$\text{DID}=\frac{\begin{array}{l} \text{Consumption of a specific medicine}\\ \text{or subgroup in DDDs} \end{array}}{\begin{array}{l} (\text{Number of inhabitants})\times (\text{Number of days}\\ \text{in the period of data collection}) \end{array}}\times 1000$$ [Eqn 1]

The proportion of ABC in each AWaRe group was determined by dividing the total DID of antibiotics in the Access, Watch, Reserve or Not-recommended groups by the total DID per year using equation 2.

**Relative antibiotic consumption**


$$\text{Relative consumption}=\frac{\begin{array}{l} \text{Consumption of a specific medicine}\\ \text{or subgroup in DID} \end{array}}{\begin{array}{l} \text{Consumption of the therapeutic medicine}\\ \text{group in DID} \end{array}}\times 100\%$$ [Eqn 2]

The Access-to-Watch Index (AW-I) was calculated by dividing the number of DIDs of Access antibiotics by the number of DIDs of Watch antibiotics and presented as a ratio using equation 3.^3,8^

**Access-to-Watch index**


$$\text{Access-to-Watch Index}=\frac{\text{Number of DID of Access antibiotics}}{\text{Number of DID of Watch antibiotics}}$$ [Eqn 3]

The amoxicillin index was derived by dividing the amoxicillin and phenoxymethylpenicillin DIDs by the annual total or arithmetic mean DID and presented as a proportion according to equation 4.^21^

**Amoxicillin index**


$$\text{Amoxicillin index}=\frac{\begin{array}{l} \text{Number of DID of amoxicillin}\\ \text{and phenoxymethylpenicillin} \end{array}}{\begin{array}{l} \text{Total number or arithmetic mean}\\ \text{of J01 antibiotic group in DID} \end{array}}\times 100\%$$ [Eqn 4]

The 75% drug utilisation index (DU75%) – antibiotic substances accounting for 75% of total consumption measured in DID using equation 5 – was calculated separately for oral and parenteral formulations and shown at ATC level 5, volume in DID, the proportion of overall consumption and classified according to AWaRe classification.^20^

**Drug utilisation 75%**


$$\text{DU75\%}=\frac{\begin{array}{l} \text{Antibiotic substances accounting for}\\ \text{75\% of total consumption in DID} \end{array}}{\begin{array}{l} \text{Total number or arithmetic mean of}\\ \text{J01 antibiotic group in DID} \end{array}}\times 100\%$$ [Eqn 5]

The compound annual growth rate (CAGR) of different AWaRe antibiotic groups, indices and antibiotic agents at ATC level 5 was estimated to highlight variations in ABC rates throughout the study period using equation 6.

**Change in consumption using CAGR**


$$\text{CAGR}={\left(\frac{\text{Value final}}{\text{Value begin}}\right)}^{1/\text{time}}-1$$ [Eqn 6]

### Ethical considerations

Ethical approval for the study was obtained from the Health Research Ethics Committee (HREC) of the North-West University (clearance number: NWU-00312-20-A1). Access permission to data sources was granted by the Head of the Limpopo Department of Health (LP-202003-012) and the Limpopo Province Pharmaceutical Depot (LPPD) manager.

## Results

### Absolute and proportional antibiotic consumption by aWaRe classification

**FIGURE 1:**
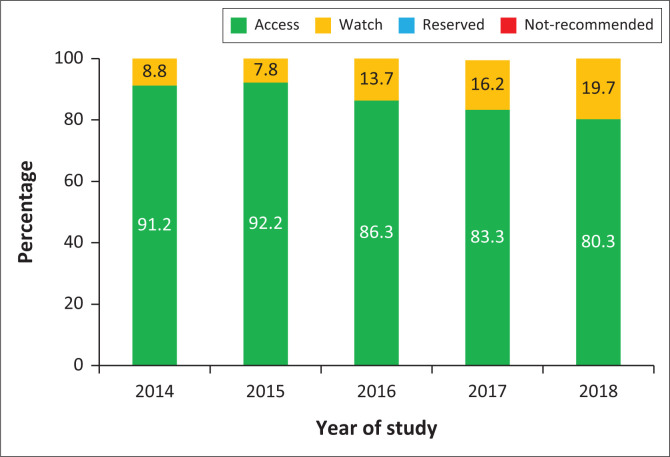
Relative antibiotic consumption by Access, Watch and Reserve classification.

A comparative analysis of ABC using the WHO AWaRe classification is shown in Figure 1. The relative consumption of Access antibiotics was above 80.0% during the study period (2014–2018). The relative consumption of the Watch antibiotics ranged from 7.8% in 2015 to 19.7% in 2018. The relative consumption of the Reserve antibiotic group was below 1.0% in all the years of the study period. There was no consumption of the Not-recommended antibiotics.

The absolute ABC according to the AWaRe classification of the WHO is presented in Table 1. The CAGR of the Access antibiotics decreased by 4.0%, from 3.8 DID in 2014 to 3.0 DID in 2018. The Watch antibiotics’ CAGR increased by 15.8%, from 0.4 DID in 2014 to 0.8 DID in 2018.

**TABLE 1:** Absolute antibiotic consumption by Access, Watch and Reserve classification.

AWaRe classification in DID	2014	2015	2016	2017	2018	Mean	s.d.	CAGR (%)
Access	3.8	4.300	3.7	5.3	3.0	4.0	± 0.9	−4.0
Watch	0.4	0.400	0.6	1.0	0.8	0.6	± 0.3	15.8
Reserve	0.0	0.001	0.0	0.0	0.0	0.0	± 0.0	0.0
Not-recommended	0.0	0.000	0.0	0.0	0.0	0.0	± 0.0	0.0

**Total DID**	**4.1**	**4.700**	**4.3**	**6.3**	**3.8**	**4.6**	**± 1.0**	**−1.6**

Note: As a result of small numbers, the DID for Reserve group in 2015 is given to three points after the decimal.

AWaRe, Access, Watch, Reserve; CAGR, compound annual growth rate; DID, DDD per 1000 inhabitants per day; s.d. standard deviation.

### The Access-to-Watch index

Table 2 presents the AW-I during the study period. The index decreased with a CAGR of 17.0% from 10.4 in 2014 to 4.1 in 2018.

**TABLE 2:** Access-to-Watch index.

Index	2014	2015	2016	2017	2018	Mean	s.d.	CAGR (%)
Access-to-Watch index	10.4	11.8	6.3	5.2	4.1	7.6	± 3.4	−17.0

s.d., standard deviation; CAGR, compound annual growth rate.

### Amoxicillin index

Both the oral amoxicillin and amoxicillin and phenoxymethylpenicillin indices are presented in Table 3. The absolute oral AI increased from 0.7 DID (17.8%) of the total ABC in 2015 to 1.6 DID (42.0%) in 2018, with a CAGR of 16.8%. Similarly, the oral amoxicillin and phenoxymethylpenicillin index increased from 1.1 DID (26.6%) in 2014 to 1.9 DID (50.6%) of the total ABC, with a CAGR of 12.0%.

**TABLE 3:** Absolute and relative amoxicillin index.

Amoxicillin index	2014		2015		2016		2017		2018		Mean	s.d.	CAGR (%)
	DID	%	DID	%	DID	%	DID	%	DID	%			
Oral amoxicillin index	0.7	17.9	1.3	27.0	1.3	31.5	2.2	33.9	1.6	42.0	1.4	± 0.5	16.8
Oral amoxicillin/phenoxymethylpenicillin	1.1	26.6	1.7	36.9	1.5	35.3	2.9	46.2	1.9	50.6	1.8	± 0.7	12.0

DID, defined daily dose per 1000 inhabitants per days; s.d., standard deviation; CAGR, compound annual growth rate.

### Drug utilisation 75% by oral route and aWaRe classification

The oral route of administration DU75% with AWaRe classification is presented in Table 4. The oral DU75% between 2014 and 2018 comprised three Access antibiotics (amoxicillin, sulphamethoxazole and trimethoprim [SMX-TMP] and doxycycline). During the first two years (2014 and 2015) of the study, the DU75% of the oral route comprised four Access antibiotics. In 2016 and 2018, the oral DU75% consisted of two Access antibiotics and one Watch antibiotic.

**TABLE 4:** Commonly consumed antibiotic at Anatomical Therapeutic Chemical classification system level 5 by absolute and relative oral route (DU75%) and Access, Watch and Reserve classification.

Oral AWaRe antibiotics	Oral route										DID		
	2014		2015		2016		2017		2018		Mean	s.d.	%
	DID	%	DID	%	DID	%	DID	%	DID	%			
**Access antibiotics**													
Sulphamethoxazole and trimethoprim	1.3	31.2	1.6	34.0	1.4	34.0	1.6	25.4	0.9	22.6	1.4	± 0.3	29.6
Doxycycline	0.9	22.8	0.6	12.8	-	-	-	-	-	-	0.8	± 0.2	16.7
Amoxicillin	0.7	17.9	1.3	27.0	1.3	31.5	2.1	33.9	1.6	41.9	1.4	± 0.5	30.8
Phenoxymethylpenicillin	0.4	8.7	0.5	9.9	-	-	0.8	12.3	-	-	0.5	± 0.2	11.6
**Watch antibiotics**													
Azithromycin	-	-	-	-	0.5	11.3	0.9	13.4	0.7	17.3	0.7	± 0.2	14.4
**Consumption DU75%**	3.3	80.5	3.9	83.7	3.3	76.8	5.4	85.0	3.1	81.9%	3.7	± 1.0	76.4

**Total DID**	**4.0**	**-**	**4.6**	**-**	**4.2**	**-**	**6.3**	**-**	**3.8**	**-**	**4.6**	**± 1.0**	**-**

s.d., standard deviation; DID, defined daily dose per 1000 inhabitants per day; DU75%, drug utilisation 75%; AWaRe, Access, Watch, and Reserve.

### Drug utilisation 75% by the parenteral route and aWaRe classification

The parenteral DU75% between 2014 and 2018 comprised one Watch antibiotic (ceftriaxone) and two Access antibiotics (metronidazole and benzylpenicillin) (Table 5). In 2014, parenteral route DU75% comprised eight Access antibiotics and two Watch antibiotics. In 2015, 2016 and 2018, metronidazole (Access antibiotic) and ceftriaxone (Watch antibiotic) constituted DU75% of the parenteral route. In 2017, the DU75% comprised benzylpenicillin (Access antibiotic) and ceftriaxone (Watch antibiotic).

**TABLE 5:** Commonly consumed antibiotic at Anatomical Therapeutic Chemical classification system level 5 by absolute and relative parenteral route (drug utilisation 75%) and Access, Watch and Reserve classification.

Parenteral AWaRe antibiotics	Parenteral route										DID		
	2014		2015		2016		2017		2018		Mean	s.d.	%
	DID	%	DID	%	DID	%	DID	%	DID	%			
**Access antibiotics**													
Metronidazole	0.0180	25.7	0.0160	44.3	0.0047	65.6	-	-	0.0110	35.5	0.0124	± 0.006	26.9
Ampicillin	0.0077	11.0	-	-	-	-	-	-	-	-	0.0077	-	16.7
Benzylpenicillin	0.0064	9.1	-	-	-	-	0.0078	13.7	-	-	0.0078	-	16.9
Benzathine benzylpenicillin	0.0062	8.9	-	-	-	-	-	-	-	-	0.0100	-	13.4
Amoxicillin + clavulanic acid	0.0009	1.2	-	-	-	-	-	-	-	-	0.0000	-	1.9
Cloxacillin	0.0008	1.1	-	-	-	-	-	-	-	-	0.0000	-	1.7
Gentamicin	0.0007	1.1	-	-	-	-	-	-	-	-	0.0000	-	1.6
Cefazolin	0.0004	0.6	-	-	-	-	-	-	-	-	0.0000	-	0.9
**Watch antibiotics**													
Ceftriaxone	0.0099	14.1	0.0130	36.0	0.0240	12.8	0.0380	66.7	0.0130	41.9	0.0200	± 0.012	42.4
Kanamycin	0.0019	2.7	-	-	-	-	-	-	-	-	0.0000	-	4.1
**Consumption DU75%**	0.0500	75.6	0.0290	80.3	0.0240	78.4	0.0458	80.4	0.0240	77.42	0.0351	± 0.013	86.3

**Total DID**	**0.07**	**-**	**0.0361**	**-**	**0.0366**	**-**	**0.057**	**-**	**0.0310**	**-**	**0.0500**	**± 0.017**	**-**

s.d., standard deviation; DID, defined daily dose per 1000 inhabitants per day; DU75%, drug utilisation 75%; AWaRe, Access, Watch, and Reserve.

### Change in consumption at ATC classification system level 5, relative consumption, compound annual growth rate and aWaRe classification

The change in consumption at ATC level 5 computed by CAGR and the 2021 AWaRe category for each agent are shown in Table 1-A1. The study included 37 antibiotic agents, 16 classified as Access, 20 as Watch, one as Reserve (linezolid) and none as Not-recommended. By 2018 (the end year of the study), four Access antibiotics were no longer being distributed, resulting in a CAGR of −100.0%. The distribution of seven Access antibiotics decreased. There was an increase in the consumption of four Access antibiotics, with cephalexin experiencing the highest increase at a CAGR of 133.7%.

Ten Watch antibiotics were no longer being distributed by 2018, resulting in a CAGR of −100.0%. Three additional Watch antibiotics have seen their consumption rates decline. The consumption of four Watch antibiotics (azithromycin, ceftriaxone, ciprofloxacin and meropenem) increased at a CAGR of 53.5%, 5.6%, 19.6% and 49.3%, respectively.

## Discussion

In 2019, the WHO proposed a global monitoring indicator requiring that by 2023, 60% of all antibiotics consumed come from the Access category, including antibiotics with the lowest risk of resistance.^2^ In this study, although the absolute consumption of Access antibiotics decreased by 4% from 3.7 DID in 2014 to 3.0 DID in 2018, relative consumption remained above 80% in each of the five years studied. The high proportion of Access antibiotics in this research could be ascribed to the procurement of large quantities of SMX-TMP (more than 50% of antibiotic sales in 2015) for medical prophylaxis against opportunistic infections in conjunction with the antiretroviral therapy.^22^

There was an increase in the consumption of the Watch antibiotics with a CAGR of 15.8%. The increase in the consumption of the Watch antibiotics could be attributed to the shortage of Access antibiotics, particularly narrow-spectrum penicillin, during the study period,^23^ which may have resulted in prescribers switching to broad-spectrum Watch antibiotics. In addition, the revisions to South Africa’s sexually transmitted infection management guidelines of 2018 promote the use of Watch antibiotics such as azithromycin and ceftriaxone ahead of Access antibiotics such as doxycycline.^24^ The increase in the consumption of Watch antibiotics and the decline in the consumption of Access antibiotics is mirrored further by the decrease in the AW-I throughout the study.

During the study period, Limpopo province had a mean AW-I of 7.7, which was higher than the country’s AW-I of 4.6 in 2015 and the global median of 1.6 based on data from 76 countries in 2015.^2^ The difference between the Limpopo province AW-I and national data could be explained by the fact that national data covered both public and private sectors, while this study’s data included only the public sector. In comparison to the public sector, there is a low level of adherence to national EML prescribing guidelines in the private sector. Consequently, there is limited cognisance of the AWaRe classification.^25^ Similar to national data, the high AW-I in our study in 2014 and 2015 (Table 3) may have been influenced by large-scale procurement of SMX-TMP from 2015 to 2017.^22^ The AW-I began declining in 2016 with the adoption of the universal HIV testing and treatment programme in conformance with international guideline recommendations.^26,27^ South African data, including both public and private sectors for an oral antibiotic formulation for young children, on the other hand, reported an AW-I of 7.0, which is comparable to the present study.^8^

According to data on oral antibiotic formulations for young children, the global consumption rate of Not-recommended antibiotics was less than 3% in 2015 and 2.4% in South Africa.^8^ The present study’s analysis found no evidence of Not-recommended antibiotic use in Limpopo province’s public sector between 2014 and 2018. The absence of Not-recommended antibiotics in this study could be explained by a strict and centralised public sector procurement approach that requires adherence to the national EML (modelled after the WHO’s EML).^28,29^

There was an increase in the consumption of Watch antibiotics and a decline in the Access antibiotics proportion and the AW-I; however, the oral AI – a complementary indicator of Access antibiotics – increased. Throughout the study period, the consumption of both amoxicillin-defined AI and amoxicillin and phenoxymethylpenicillin-defined AI increased at a CAGR of 16.8% and 12.0%, respectively. In 2015, the global median amoxicillin and phenoxymethylpenicillin index was 18.5%.^2^ In comparison, the South African AI was higher than the global median in 2015, at 21.6% using the same criteria.^3^ However, using the original definition of amoxicillin as a proportion of total consumption of J01 systematic antibiotics,^21^ the global mean for oral antibiotic formulations for young children was 30.0%. In comparison, the AI for South Africa was 50.4%.^8^ The AI was created to promote the development of initiatives to increase access to medicine.^2^ The high amoxicillin AI found in the present study is aligned with the South African standard treatment guidelines and pneumonia guidelines,^29,30^ similar to trends in treating respiratory tract infections in other upper-middle-income African countries, such as Algeria, where health authorities promote prescribing based on national guidelines.^2^

This study showed an increase in the relative consumption of Watch antibiotics (Figure 1), consistent with the global study and national report findings.^2,22^ The increase in Watch antibiotics in the present study could be attributed to an increase in azithromycin, ceftriaxone, ciprofloxacin and meropenem consumption (Table 1-A1). The increased consumption of azithromycin may perpetuate macrolide-resistant *Streptococcus pneumoniae* and non-macrolide-resistant antibiotics from the Access group (aminoglycosides, beta-lactams, trimethoprim and metronidazole).^31^ High consumption of ceftriaxone could accelerate the development and spread of third-generation cephalosporin-resistant Enterobacterales and *Neisseria gonorrhoeae*. The increasing use of ciprofloxacin is associated with fluoroquinolone-resistant *N. gonorrhoeae, Shigella* spp., *Campylobacter* and *Salmonella* spp. The high consumption of meropenem is associated with carbapenem-resistant *Acinetobacter baumannii, Pseudomonas aeruginosa* and Enterobacteriaceae.^32^ The only Reserve antibiotic consumed in Limpopo province between 2014 and 2018 was linezolid. The main rationale for its use in the public sector is for the management of multi-drug-resistant tuberculosis.^33^

The policy, practice and research recommendations of this study are that it should establish a baseline for ABC in the public sector of the Limpopo province using internationally comparable indicators. To keep track of ABC, the province should consider adopting the GLASS methodology and AWaRe classification in its annual routine ABC surveillance system, according to the WHO’s strategic priorities for combating ABR and in line with the existing South African Antimicrobial Resistance National Strategy Framework: a One-Health approach 2017–2024, and guidelines for the prevention and containment of antimicrobial resistance in South African hospitals.^34,35,36^ This would entail quantifying ABC, monitoring provincial antibiotic policies and evaluating ASP activities using the AWaRe classification. The PDoH should consider setting a target to maintain their performance on Access antibiotics consumption (above the global target of 60.0%) and AI (between 30.0% and 50.0%) while concurrently implementing ASP activities to mitigate against the increasing Watch antibiotics consumption and AW-I.

This study identified four antibiotics in the Watch group (azithromycin, ceftriaxone, ciprofloxacin and meropenem) increasing in consumption. This may require additional research to identify the determinants of increased consumption. Policymakers may activate ASP activities to evaluate the appropriate use of the identified Watch antibiotics at patient level. Furthermore, policymakers should encourage and support ASP activities (via leadership and supply of resources such as funding, training and time). Administratively, it is necessary to ensure Access antibiotics’ continued availability and accessibility, as stock-outs may force prescribers to use Watch antibiotics. Lastly, two AI definitions were identified: the AI as a proportion of oral amoxicillin relative to the total consumption of J01 systemic antibiotics^21^ or AI defined as the proportion of oral amoxicillin and phenoxymethylpenicillin relative to the total J01 systemic antibiotics.^2^ Consensus on an AI definition may minimise the likelihood of misinterpretation by researchers and policymakers.

This study had the following limitations: firstly, the ATC and DDD method has inherent limitations, as it is primarily a proxy for actual antibiotic use, and DDDs are constantly revised, necessitating careful interpretation, particularly when comparing data. Secondly, this study estimated ABC based on sales data, which may include antibiotics distributed to facilities but not utilised. However, sales data are closer to actual utilisation than other data sources such as purchase data.^19^ Thirdly, the study relied entirely on public sector data; the findings of this study cannot therefore be generalised to the Limpopo province overall. As it was not possible to determine the population size for the public sector alone, the ABC rate denominators used were the total provincial population, which included both public and private sectors. This may lead to the underestimation of ABC in Limpopo province. Future studies should consider using national or provincial estimates of the population served by either the private or the public sector as a proxy to mitigate the effect of an inseparable population under surveillance to which the data apply to reduce the underestimation of ABC caused by aggregated denominators.^17,19^ Fourthly, it was not possible to disaggregate the data into the two health care levels (hospital and community) within the public sector because the data source did not permit such a provision. Particularly, outpatient hospital care ABC could not be separated from hospital care ABC because the former is part of the community sector. As the ABC’s data sources evolve and data collectors and providers gain experience, this may become possible in the future.^19^ Lastly, the study used ABC data based on data collected over time from distribution warehouse sales database. The inherent limitations of sales data are that they do not comprise information about individual patient details (antibiotic use). Furthermore, the ABC sales data were not linked to ecological ABR surveillance data to enable causality association assessment. Nevertheless, the ABC methodology is standardised and reliably allows for international comparability and simple integration into national or local ABC surveillance systems.^19^

In conclusion, the Limpopo province public sector met the global monitoring indicator, with antibiotic use in the Access group exceeding 80% during the study period, decreasing by 4.0% CAGR. However, the 15.8% CAGR increase in the consumption of Watch antibiotics and 17.0% decrease in AW-I are a warning signal. It needs to be addressed by policymakers, clinicians and researchers as part of a coordinated global effort to combat the growing global threat posed by ABR.

## Appendix 1

**TABLE 1-A1:** Absolute antibiotic consumption at Anatomical Therapeutic Chemical classification system level 5, compound annual growth rate and aWaRe classification.

Item description	2014 DIDs	2015 DID	2016 DIDs	2017 DIDs	2018 DIDs	CAGR (%)	AWaRe category
Doxycycline	0.934000	0.600000	0.295000	-	-	−100.0	Access
Flucloxacillin	0.286000	0.230000	0.252000	0.445000	-	−100.0	Access
Gentamicin	0.000740	0.001600	0.000260	0.000410	-	−100.0	Access
Amikacin	0.000056	-	-	0.000071	-	−100.0	Access
Ampicillin	0.007700	0.000120	0.000710	0.000025	0.00000095	−83.5	Access
Benzylpenicillin	0.006400	0.002500	0.003900	0.007800	0.0014000	−26.2	Access
Cloxacillin	0.000770	0.000600	0.000036	0.001200	0.000200	−23.6	Access
Metronidazole	0.018000	0.016000	0.004700	0.004600	0.011000	−9.4	Access
Benzathine benzylpenicillin	0.006200	0.001700	0.001400	0.003500	0.003900	−8.9	Access
Sulphamethoxazole and trimethoprim	1.278000	1.587000	1.443000	1.608000	0.857000	−7.7	Access
Phenoxymethylpenicillin	0.357000	0.460000	0.163000	0.780000	0.327000	−1.7	Access
Nitrofurantoin	-	-	-	0.000150	-	0.0	Access
Cefazolin	0.000430	0.000016	0.000052	0.000570	0.000850	14.6	Access
Amoxicillin	0.732000	1.262000	1.334000	2.145000	1.589000	16.8	Access
Amoxicillin/enzyme inhibitor	0.110000	0.148000	0.161000	0.312000	0.250000	17.8	Access
Cephalexin	0.0000043	0.000018	-	0.000140	0.000300	133.7	Access
Linezolid	-	0.000720	-	-	-	0.0	Reserve
Erythromycin	0.204000	0.060000	0.002000	-	-	−100.0	Watch
Cefixime	0.017000	0.009100	0.000190	-	-	−100.0	Watch
Moxifloxacin	0.014000	0.008300	-	0.001800	-	−100.0	Watch
Levofloxacin	0.003500	0.001400	0.000240	0.002300	-	−100.0	Watch
Cefoxitin	0.000170	-	-	-	-	−100.0	Watch
Cefotaxime	0.000160	0.0000024	0.000130	0.000040	-	−100.0	Watch
Vancomycin	0.000024	0.0000024	-	0.000160	-	−100.0	Watch
Clindamycin	0.000054	0.000068	-	0.000028	-	−100.0	Watch
Cefuroxime	0.000036	-	-	-	-	−100.0	Watch
Cefepime	0.000019	-	-	-	-	−100.0	Watch
Piperacillin + tazobactum	0.0000064	0.0000024	-	-	0.00000024	−48.1	Watch
Imipenem + cilastatin	0.000091	0.000051	0.000076	0.000025	0.000011	−34.5	Watch
Kanamycin	0.001900	0.000190	0.001300	0.000470	0.000910	−13.7	Watch
Ofloxacin	-	0.001500	-	-	-	0.0	Watch
Clarithromycin	-	0.000440	-	0.000660	-	0.0	Watch
Ceftazidime	-	-	0.0000024	0.0000024	-	0.0	Watch
Ceftriaxone	0.009900	0.013000	0.024000	0.038000	0.013000	5.6	Watch
Ciprofloxacin	0.031000	0.047000	0.074000	0.13200	0.076000	19.6	Watch
Meropenem	0.0000054	-	-	0.000038	0.000040	49.3	Watch
Azithromycin	0.077000	0.222000	0.479000	0.848000	0.657000	53.5	Watch

DID, defined daily dose per 1000 inhabitants per day; CAGR, compound annual growth rate; AWaRe, Access, Watch and Reserved.
